# Supplementary material for: Empirical methods for controlling false positives and estimating confidence in ChIP-Seq peaks
Source: BMC Bioinformatics. 2008 Dec 5;9:523. doi: 10.1186/1471-2105-9-523 (PMC2628906; doi:10.1186/1471-2105-9-523)
Supplement: Additional File 1 — A variety of html documents from the USeq web site detailing the available applications, their best usage, output file type descriptions, command line menus, etc. [file 1471-2105-9-523-S1.zip › USeqUserGuides/JavaDoc.zip!/JavaDoc/cgar/beans/UserBean.html]

UserBean


|  |  |  |
| --- | --- | --- |
| **Overview** \| **Package** \| **Class** \| **Use** \| **Tree** \| **Deprecated** \| **Index** \| **Help** | |  |
| **PREV CLASS**   **NEXT CLASS** | **FRAMES**    **NO FRAMES**     **All Classes** |  |
| SUMMARY: NESTED \| FIELD \| CONSTR \| METHOD | DETAIL: FIELD \| CONSTR \| METHOD |  |


---


## cgar.beans Class UserBean

```
java.lang.Object
  cgar.beans.UserBean
```

---

``` public class UserBean extends java.lang.Object ```

User info, where possible this should come from GNomEx, use U ids/ passwords, when not available store
md5 salted encrypted passwords, do not use db specific encryption function, use Nix's MD5Crypt.java class.

---

**Constructor Summary**

`UserBean()`

`UserBean(Util util,
LoginBean loginBean)`


| **Method Summary** | |
| --- | --- |
| `java.lang.String` | `getDateLastLogin()` |
| `java.lang.String` | `getEmail()` |
| `java.lang.String` | `getFirstName()` |
| `int` | `getId()` |
| `LabGroupUserType[]` | `getLabGroupUserTypes()` |
| `java.lang.String` | `getLastName()` |
| `java.lang.String` | `getLoginName()` |
| `boolean` | `isGuest()` |
| `boolean` | `loadRemainingFields()`             Loads |
| `boolean` | `makeLabGroupPermissions()`             Finds available lab groups associated with this user. |
| `void` | `setDateLastLogin(java.lang.String dateLastLogin)` |
| `void` | `setEmail(java.lang.String email)` |
| `void` | `setFirstName(java.lang.String firstName)` |
| `void` | `setGuest(boolean guest)` |
| `void` | `setId(int id)` |
| `void` | `setLabGroupUserTypes(LabGroupUserType[] labGroupUserTypes)` |
| `void` | `setLastName(java.lang.String lastName)` |
| `void` | `setLoginName(java.lang.String loginName)` |

**Methods inherited from class java.lang.Object**

`equals, getClass, hashCode, notify, notifyAll, toString, wait, wait, wait`

**Constructor Detail**

### UserBean

```
public UserBean()
```

---


### UserBean

```
public UserBean(Util util,
                LoginBean loginBean)
```


**Method Detail**

### loadRemainingFields

```
public boolean loadRemainingFields()
```

:   Loads

---


### makeLabGroupPermissions

```
public boolean makeLabGroupPermissions()
```

:   Finds available lab groups associated with this user.

---


### getDateLastLogin

```
public java.lang.String getDateLastLogin()
```

---


### setDateLastLogin

```
public void setDateLastLogin(java.lang.String dateLastLogin)
```

---


### getEmail

```
public java.lang.String getEmail()
```

---


### setEmail

```
public void setEmail(java.lang.String email)
```

---


### getFirstName

```
public java.lang.String getFirstName()
```

---


### setFirstName

```
public void setFirstName(java.lang.String firstName)
```

---


### getId

```
public int getId()
```

---


### setId

```
public void setId(int id)
```

---


### getLastName

```
public java.lang.String getLastName()
```

---


### setLastName

```
public void setLastName(java.lang.String lastName)
```

---


### getLoginName

```
public java.lang.String getLoginName()
```

---


### setLoginName

```
public void setLoginName(java.lang.String loginName)
```

---


### isGuest

```
public boolean isGuest()
```

---


### setGuest

```
public void setGuest(boolean guest)
```

---


### getLabGroupUserTypes

```
public LabGroupUserType[] getLabGroupUserTypes()
```

---


### setLabGroupUserTypes

```
public void setLabGroupUserTypes(LabGroupUserType[] labGroupUserTypes)
```


---


|  |  |  |
| --- | --- | --- |
| **Overview** \| **Package** \| **Class** \| **Use** \| **Tree** \| **Deprecated** \| **Index** \| **Help** | |  |
| **PREV CLASS**   **NEXT CLASS** | **FRAMES**    **NO FRAMES**     **All Classes** |  |
| SUMMARY: NESTED \| FIELD \| CONSTR \| METHOD | DETAIL: FIELD \| CONSTR \| METHOD |  |


---
